# Detection of sexually transmitted pathogens and co-infection with human papillomavirus in women residing in rural Eastern Cape, South Africa

**DOI:** 10.7717/peerj.10793

**Published:** 2021-03-03

**Authors:** Ongeziwe Taku, Adrian Brink, Tracy L. Meiring, Keletso Phohlo, Charles B. Businge, Zizipho Z.A. Mbulawa, Anna-Lise Williamson

**Affiliations:** 1 Department of Pathology, Faculty of health sciences, University of Cape Town, Cape Town, South Africa; 2 Institute of Infectious Disease and Molecular Medicine, University of Cape Town, Cape Town, Western Cape, South Africa; 3 Division of Medical Microbiology, Department of Pathology, Faculty of Health Sciences, University of Cape Town, Cape Town, South Africa; 4 Department of Obstetrics and Gynaecology, Nelson Mandela Academic Hospital, Mthatha, South Africa; 5 Department of Obstetrics and Gynaecology, Faculty of Health Sciences, Walter Sisulu University, Mthatha, South Africa; 6 SAMRC Gynaecological Cancer Research Centre, University of Cape Town, Cape Town, South Africa; 7 Department of Laboratory Medicine and Pathology, Walter Sisulu University, Mthatha, South Africa; 8 National Health Laboratory Service, Nelson Mandela Academic Hospital, Mthatha, South Africa

**Keywords:** HPV, Sexually transmitted pathogens, *Treponema pallidum*, *Trichomonas Vaginalis*, *Mycoplasma genitalium*, *Mycoplasma hominis*, *Ureaplasma*

## Abstract

**Background:**

South African women of reproductive age have a high burden of sexually transmitted infections (STIs), including human papillomavirus (HPV) infection. However, there is limited information on the prevalence of sexually transmitted pathogens in women from rural Eastern Cape Province, South Africa. The study aims at determining the prevalence of sexually transmitted pathogens and co-infection with high-risk (HR) HPV among women from rural Eastern Cape Province, South Africa.

**Methods:**

A total of 205 cervical specimens were collected from women aged ≥ 30 years from a rural community-based clinic. The samples were tested for a panel of pathogenic STIs [*Chlamydia trachomatis* (serovars A-K & L1-L3), *Haemophilus ducreyi*, Herpes Simplex Virus (Types 1 & 2), *Neisseria gonorrhoeae*, *Treponema pallidum*, *Trichomonas vaginalis* (*TV*), and pathobionts [*Mycoplasma genitalium (MG), Mycoplasma hominis* (*MH*) and *Ureaplasma* spp. (*UP*)] using a multiplex PCR STD direct flow chip assay through a manual Hybrispot platform (Master Diagnostica, Granada, Spain). HR-HPV detection was performed by Hybrid Capture-2 assay.

**Results:**

High-risk HPV prevalence was 32.2% (66/205) and HIV-1 prevalence was 38.5% (79/205). The overall prevalence of six pathogenic STIs was 22.9% (47/205), with *TV* having the highest prevalence (15.6%; 32/205). UP (70.2%, 144/205) and *MH* (36.6%, 75/205) were the most frequently detected pathobionts. Co-infection with ≥ 2 pathogens pathobionts was observed among 52.7% (108/205) participants. Of the six pathogenic STIs, three participants had more than one STI (1.46%) with the presence of *MH* and *UP*. HSV-2 (OR: 4.17, CI [1.184–14.690]) and HIV infection (OR: 2.11, CI [1.145–3.873]) were independent STIs associated with HR-HPV infection.

**Conclusions:**

The high prevalence of pathogenic STIs underscores the need to improve syndromic management policy by implementing effective strategies of prevention, screening tests, and management. HSV-2 and HIV positive remain strongly associated with HR-HPV infection.

## Introduction

Internationally, sexually transmitted infections (STIs) are a significant public health problem, with an estimated more than one million people infected each day. According to the World Health Organization, the global estimate of new infections with commonly treatable STIs [*Trichomonas vaginalis* (*TV*)*, Treponema pallidum* (*TP*)*, Chlamydia trachomatis* (*CT*) and *Neisseria gonorrhoeae* (*NG*)] was 376.4 million in 2016 (WHO, 2018). Africa accounts for 69 million new infections of treatable STIs with women having a high burden of *TV* (11, 8%) and *CT* (5, 0%) (WHO, 2018). STIs have an impact on women’s health, associated with cervicitis, urethritis, pelvic inflammation, complications of reproductive health, and poor pregnancy outcomes (Mermelstein & Plax, 2016). South African women have a high prevalence of STIs, ranging from 12.7% to 47.8%, which differs by age, region, and population (Joseph Davey et al., 2019; Moodley et al., 2015; Mudau et al., 2018; Naidoo et al., 2014). The burden of STIs is more common among women of reproductive age (15–49 years) (WHO, 2018). The acquisition of new STIs occurs at all ages, and high rates of STIs are more likely to be found in younger women (Naidoo et al., 2014). In South African studies, women age <25 years are almost 2-fold more likely to have STIs compared to older women (>25 years) and have high rates of co-infections (Mbulawa et al., 2018; Menezes et al., 2018; Naidoo et al., 2014).

Human papillomavirus (HPV) is the most common and infectious viral STI, with 291 million new infections estimated to have occurred in 2016, with a particularly high burden among women in Southern Africa (Bruni et al., 2010; WHO, 2018). Most HPV infections are transient, but 5% remain persistent and can progress to high-grade lesions or cervical cancer (Schiffman et al., 2011). The high burden of HPV infection is influenced by several factors, including co-infection with other STIs. HIV infection is a significant independent factor of HPV, and infection with either HPV or HIV is thought to enhance the spread of the other infection (Smith-McCune et al., 2010). HIV-positive women have been reported to have a higher prevalence and higher viral load of high-risk (HR) HPV compared to HIV-negative women (Taku et al., 2020). In addition, *CT*, Herpes Simplex Virus-2 (HSV-2), and *NG* increase the risk of HIV acquisition (Adachi et al., 2015; Johnson & Lewis, 2008; Looker et al., 2017). These STIs are co-factors of HPV and may have an impact on the natural history of HPV (Deluca et al., 2011; De Abreu et al., 2016; Paba et al., 2008; Smith et al., 2002a; Smith et al., 2002b). The association of HPV infection with some of these STIs is due to chronic inflammation or immunosuppression, which promotes the susceptibility to and progression of HR-HPV persistent infection (Adefuye & Sales, 2012; Denny et al., 2012; Liu et al., 2016; Paba et al., 2008; Silins et al., 2005). HPV-positive women co-infected with one or more of these STIs are at high risk of developing cervical cancer diseases and invasive cervical cancer (Deluca et al., 2011; Paba et al., 2008; Smith et al., 2002a). Consequently, women harbouring or having a history of *CT* are less likely to clear HPV infection, and two times more likely to develop cervical cancer diseases (Jensen et al., 2014; Lehtinen et al., 2011; Vriend et al., 2015). Moreover, sexually transmitted pathobionts such as *Mycoplasma hominis* (*MH*) and *Ureaplasma spp.* (*UP*) are associated with an increased risk of HPV persistent infection and abnormal cervical cytology (Parthenis et al., 2018). Therefore, it is crucial to screen for these sexually transmitted pathogens to reduce the risk of transmission and their outcome.

In South Africa, the current strategy for diagnosing STIs is through clinical indicators such as vaginal discharge, pelvic pain, and ulcerative genital lesions (Health NDo, 2015). The syndromic management approach has been successful in treating many pathogens causing STIs and reducing the burden of other STIs such as *TP* (Kularatne et al., 2018). However, this approach may result in overtreatment, antimicrobial resistance, and is not effective in people with STIs who do not show any clinical symptoms (Mayaud & Mabey, 2004). South African studies reported a high prevalence of asymptomatic women, ranging from 50–75%, harbouring genital tract infections (Francis et al., 2018; Wilkinson et al., 1999). Since many STIs are asymptomatic and missed by the syndromic management approach, laboratory-based diagnosis remains the only strategy that allows the detection of genital tract infections (Sznitman et al., 2010).

Moreover, screening programmes allow people to be informed about STIs, which helps to prevent and manage the spread of STIs (Sznitman et al., 2010). Women residing in rural areas have limited knowledge of STIs and not likely to be informed about STIs services due to lack of access to healthcare facilities or facilities having limited resources to treat STIs (Cristillo et al., 2017; Wi et al., 2019). There is limited information on STIs and the prevalence of specific sexually transmitted pathogens in Eastern Cape Province. Therefore, the study aims at investigating the prevalence of sexually transmitted pathogens in women from rural Eastern Cape using molecular detection.

## Materials and Methods

Cohort description or description of study participants: Two hundred and five cervical samples were selected from a cross-sectional study done between September 2017 and August 2018. The cross-sectional study has been described in detail previously (Taku et al., 2020). Briefly, women aged 30 years or more attending cervical cancer screenings or for other reasons were recruited from a community-based clinic within the OR Tambo District, Eastern Cape. All signed consent forms were obtained from all enrolled women. Women were requested to test for HIV if they were not aware of their HIV status or if their HIV status was not documented on their health card. Women received pre-HIV testing counselling prior to and after testing them for HIV using a rapid test (Alere Determine™ HIV-1/2 Ag/Ab Combo, Alere, Waltham, MA). The protocol of this study was approved by the Human Research Ethics Committees of the University of Cape Town (UCT) (HREC reference 615/2017), Walter Sisulu University (reference 016/2017), and Eastern Cape Department of Health Ethics (EC reference 2017RP0_484). Cervical specimens were stored in the Digene Specimen Transport Medium (Qiagen, Inc., Gaithersburg, MD; USA), transported to UCT, and kept at −80 °C until further analysis.

There was no special selection criteria considered for this study. The median age of the women was 45 years (IQR: 38-53), and the median number of lifetime sexual partners was three. A total of 61.5% (126/205) of the women reported not using a condom during their last sexual encounter. More than half of women (58.5%, 120/205) reported not using any method of contraception with their current partner. One hundred and ten (110) study participants (53.6%) reported having had vaginal discharge with 44.5% (49/110) reporting to have occurred more or equal to six months (Table 1). 38.5% (79/205) women were positive for HIV and 96.2% (76/79) were on antiretroviral drugs. The majority of women (88.8%, 182/205 ) had normal cervical cytology while , 7.8% (16/205) were positive for ASCUS, 2.0% (4/205) for low grade intraepithelial squamous lesions and 1.0% (2/205) for high grade squamous intraepithelial lesions. One participant had had an inadequate result (0.5%,1/205).

**Table 1:** Description of the study participants.

Variables	% (n/N)
Age in years, median (IQR)	45 (38–53)
**Age category**	
30–39 years	33.7% (69/205)
40–49 years	28.8% (59/205)
≥50 years	37.6% (77/205)
**Lifetime partners**	
1	15.1% (31/205)
2	29.8% (61/205)
≥3	55.1% (113/205)
**Sexual partners past 12 months**	
0	26.3% (54/205)
≥1	73.7% (151/205)
**Sexual partners past 1 month**	
0	41.5% (85/205)
≥1	58.5% (120/205)
**Vaginal sexual intercourse**	
0	49.3% (101/205)
1–3	33.2% (68/205)
≥4	17.1% (35/205)
**Condom use**	
No	61.5% (126/205)
Yes	37.1% (76/205)
**Discharge**	
No	46.3% (95/205)
Yes	53.7% (110/205)
**Frequency of vaginal discharge**	
Current/last week	18.1% (37/205)
More than a week and less than 6 months	11.2% (23/205)
More than or equal to 6 months	23.9% (49/205)
**Using any contraception with current partner**	
No	58.5% (120/205)
Yes	40.0% (82/205)
**Pregnancy**	
No	4.4% (9/205)
Yes	95.6% (196/205)
**HIV infection**	
Negative	61.5% (126/205)
Positive	38.5% (79/205)

### DNA extraction

The DNA was extracted from each cervical specimen (400 µl) using the MagNA Pure Compact Nucleic Acid Isolation kit (Roche Diagnostic, Mannheim, Germany) on an automated Roche MagNA Pure Compact system. DNA was eluted in 100 µl elution buffer and stored at −20 °C until further use.

### Detection of sexually transmitted pathogens

Extracted DNA was used for detection of STD performed using multiplex PCR STD direct flow chip assay through a manual Hybrispot platform (Master Diagnostica, Granada, Spain) following the manufacturer’s instructions. The panel detects the following pathogens: *TP*, HSV (Type 1 & 2), *TV*, *CT* (Biovar LGV: Serovars L1-L3 & Serovars A-K), *NG*, *Haemophilus ducreyi* as well as *UP* (urealyticum/parvum), *MG* and *MH* (Barrientos-Durán et al., 2020). The image results of each chip membrane were captured by a camera, and analysis was performed automatically with HybriSoft software.

### Detection of HR-HPV infection

Cervical specimens in Digene transport medium were tested for 13 HR-HPV types (HPV16, 18, 31, 33, 35, 39, 45, 51, 52, 56, 58, 59 and 68) using the Hybrid Capture-2 (HC-2) assay (Qiagen, Inc., Gaithersburg, MD; USA) according to the manufacturer’s protocol. A ratio of relative light units/cut-off ≥1 was considered positive, while a ratio of < 1 was considered negative for HR-HPV types.

### Data analysis

Single infection was defined as being positive for one of any pathogenic STIs or pathobionts. Multiple infections was defined as having two or more microorganisms (pathogenic STIs/pathobionts). Statistical analysis was performed using STATA 15.0 (STATA Corp, College Station, TX, USA). Univariate logistic regression models were conducted to determine the association between sexually transmitted pathogens and HR-HPV infection. Multivariate analysis was done using the statistically significant variables (*p*-value < 0.05) of the univariate logistic regression models to identify the sexually transmitted pathogens that are independently associated with HR-HPV infection.

## Results

### Sexually transmitted pathogens / pathobionts and pattern of infection

**Figure 1:**
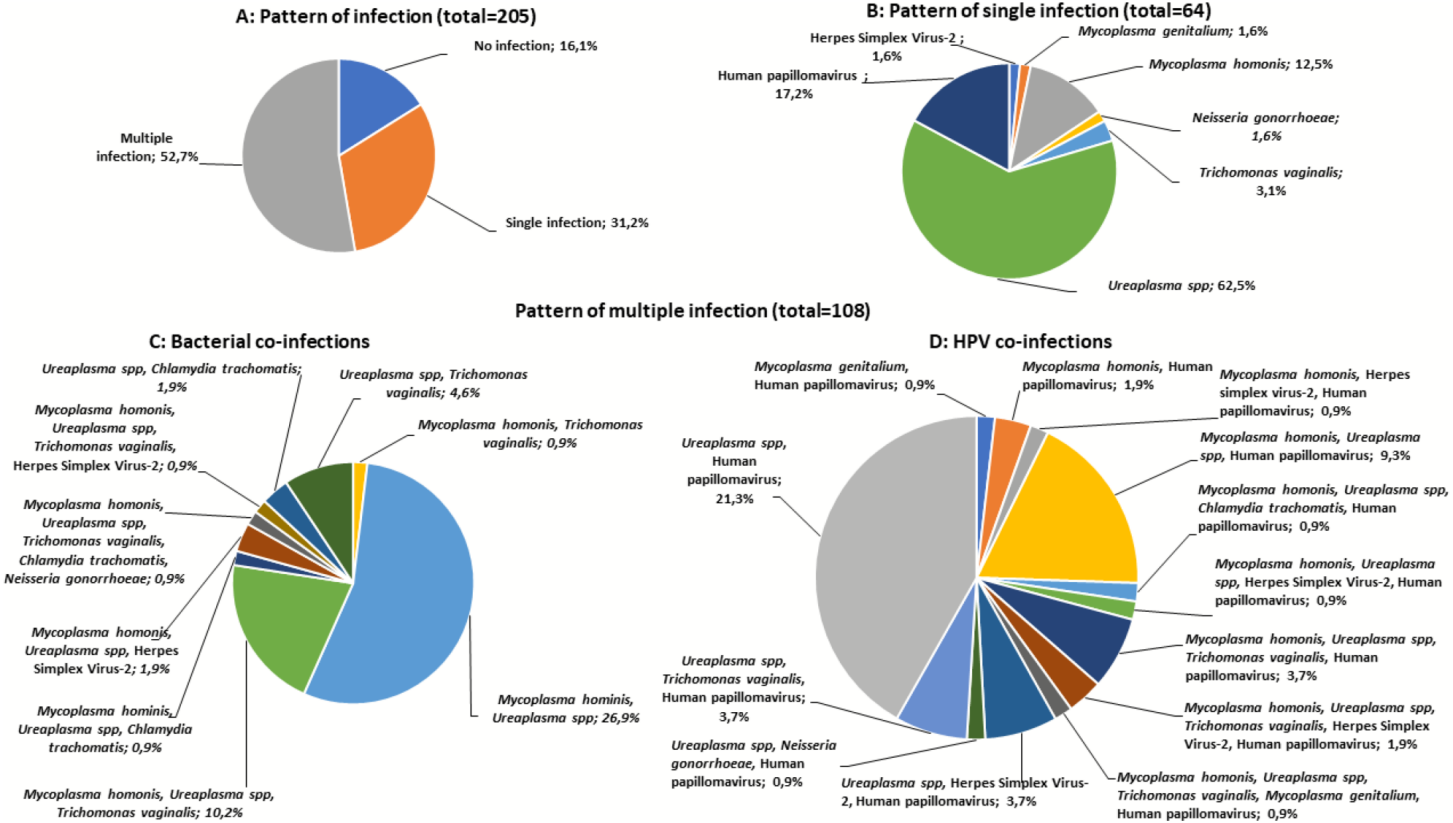
Patterns of sexually transmitted pathogens in women of rural South Africa. (A) The pattern of infections (no infection, single infection, and multiple infections) (B) The pattern of single infections in women with one of the nine sexually transmitted pathogens [*Chlamydia trachomatis* (CT), Herpes simplex virus-2 (HSV-2), *Mycoplasma genitalium* (MG), *Mycoplasma hominis* (MH), *Neisseria gonorrhoeae* (NG), *Trichomonas vaginalis* (TV), Ureaplasma spp. (UP), and Human papillomavirus (HPV)]. C&D) The pattern of co-infections in women with more than one sexually transmitted pathogens evaluated.

HR-HPV prevalence was 32.2% (66/205) and HIV-1 prevalence was 38.5% (79/205). The overall prevalence of the six STIs was 22.9% (47/205) with a highest number of women positive for *TV* (15.6%, 32/205) followed by HSV-2 (5.9%, 12/205), *CT* (2.4%, 5/205) and *NG* (1.5%, 3/205) (Table 2). Of the pathobionts, *UP* (70.2%, 144/205) and *MH* (36.6%, 75/205) were the most frequently detected (Table 2). Overall, the prevalence of single infection was 31.2% (64/205), with most women infected with *UP* (62.5%) and HR-HPV infection (17.2%) (Figs. 1A, 1B and 1C). Furthermore, multiple infections were found in 52.7% (108/205) women, with 30.7% had dual infection (2 pathogens / pathobionts) and 22.0% co-infected with more than three pathogens / pathobionts (Figs. 1B, 1C and 1D). A higher proportion of co-infection was observed among women with *UP,* whereby *UP*/*MH* (26.9%), *UP*/HPV (21.3%), and *MH*/*UP*/TV (10.2%) were the most commonly detected co-infections (Figs. 1C and 1D). Of the six pathogenic STIs, three participants had more than one STI (1.46%) with the presence of *MH* and *UP*. Women with HSV-2 infection were almost five times more likely to be infected with HR-HPV (OR: 4.65, CI [1.35–16.071]). In the multivariate analysis, HSV-2 (OR: 4.17, CI [1.184–14.690]) and HIV infection (OR: 2.11, CI [1.145–3.873]) remained the significant risk factors of HR-HPV infection (Table 3).

**Table 2:** Prevalence of sexually transmitted infections detected using multiplex PCR STD direct flow chip assay.

**Variables**	**% (n/N)**
*Chlamydia trachomatis* (serovars L1-L3)	0.5% (1/205)
*Chlamydia trachomatis* (serotypes A-K)	2.0% (4/205)
Herpes simplex virus Types I	0.0% (0/205)
Herpes simplex virus Types II	5.9% (12/205)
*Trichomonas vaginalis*	15.6% (32/205)
*Neisseria gonorrhoeae*	1.5% (3/205)
*Treponema pallidum*	0.0% (0/205)
*Haemophilus ducreyi*	0.0% (0/205)
*Mycoplasma hominis*	36.6% (75/205)
*Ureaplasmas* (*U. urealyticum* or *U. parvum*)	70.2% (144/205)
*Mycoplasma genitalium*	1.5% (3/205)

**Table 3:** Association of sexually transmitted pathogens and HR-HPV infection.

	**HR-HPV prevalence**	****	**Univariate analysis**		****	**Multivariate analysis**	
**Variables**	**% (n/N)**	****	**OR (95% Cl)**	***P*-value**		**OR (95% Cl)**	***P*-value**
*Mycoplasma hominis*							
Negative	33.9% (44/130)		Ref				
Positive	29.3% (22/75)		0.81 (0.438–1.502)	0.506			
*Ureaplasmas* species (*U. urealyticum* or *U. parvum*)							
Negative	24.6% (15/61)		Ref				
Positive	35.4% (51/144)		1.68 (0.856–3.305)	0.131			
*Trichomonas Vaginalis*							
Negative	31.8% (55/173)		Ref				
Positive	34.4% (11/32)		1.12 (0.507–2.493)	0.838			
Herpes Simplex Virus Types II							
Negative	30.1% (58/193)		Ref			Ref	
Positive	66.7% (8/12)		4.65 (1.35–16.071)	**0.015**		4.17 (1.184–14.690)	**0.026**
*Chlamydia Trachomatis* (serovars L1-L3 & A-K)							
Negative	32.5% (65/200)		Ref				
Positive	20.0% (1/5)		0.52 (0.057–4.739)	0.561			
*Neisseria gonorrhoeae*							
Negative	32.2% (65/202)		Ref				
Positive	33.3% (1/3)		1.05 (0.094–11.834)	0.966			
*Mycoplasma genitalium*							
Negative	31.7% (64/202)		Ref				
Positive	66.7% (2/3)		4.31 (0.384–48.434)	0.236			
HIV infection							
Negative	25.4% (32/126)		Ref			Ref	
Positive	43.0% (34/79)		2.22 (1.219–4.042)	**0.009**		2.11 (1.145–3.873)	**0.017**

**Notes.**

HR-HPV high-risk human papillomavirus OR odds ratio CI confidence intervals ref reference, Highlighted values; significant *p*-value

Of the women with self-reported vaginal discharge, a total of 21 (19.1%) were positive for treatable STIs, including 56.3% (18/32) with *TV* and 60.0% (3/5) with *CT* (refer to supplementary data). All women positive for *NG* self-reported not having had vaginal discharge (100.0%, 3/3). Similarly, the pelvic examination done by the study nurse showed that 28 .1% (9/32) of women positive for *TV* had vaginal discharge but none of the women positive for *NG* had vaginal discharge. The association of STIs and behavioural factors are depicted in Table S1. Women having 3 or more lifetime sexual partners was associated with STIs (OR: 3.69, CI [1.047–12.986], *P* = 0.042). In addition, women age ≥50 years (OR:0.38, CI [0.185–0.797], *P* = 0.010) or reported having had vaginal discharge for more than or equal to six months (OR:0.38, CI [0.149–0.967], *P* = 0.042) had a lower risk of HPV infections whereas three or more lifetime sexual partners (OR:3.08, CI [1.098–8.619], *P* = 0.033), HIV positive (OR:2.22, CI [1.219–4.042], *P* = 0.009) were significant risk factors of increased HR-HPV infection (Table S2). However, in the multivariate analysis none of these factors remained a significant risk factor of HR-HPV infection (Table S2).

## Discussion

We investigated the prevalence of sexually transmitted pathogens/pathobionts and co-infection with HPV infection among women from rural Eastern Cape. The study demonstrates a high overall prevalence of the conventional pathogenic STIs (22.9%) and confirms the high prevalence of *TV* (15.6%) in this population. The high burden of *TV* has been previously reported in South African women of a rural region, occurring in 66% of asymptomatic women (De Waaij et al., 2017). It has been found that hormonal changes and menstrual bleeding contribute to the increase of *TV* and put women at high risk of being more susceptible to acquisition and persistent infection (Poole & McClelland, 2013). Women with *TV* persistence have an increased risk of acquiring HIV, a high viral load of HIV, and a likelihood of transmitting HIV infection to their sexual partners (De Waaij et al., 2017; Van der Pol, 2007). Also, the high prevalence of *TV* may cause serious reproductive health problems in this group of women, as shown in previous studies (Kissinger, 2015). Therefore, better screening programmes and control measures to reduce the burden of this STI are of critical importance, particularly among asymptomatic women.

The positivity rate for *CT* (2.4%) and *NG* (1.5%) was low in this cohort and similar to that observed in community-based studies conducted among older women from rural and urban regions of sub-Saharan Africa (Dubbink et al., 2018). The highest rates of *CT*/*NG* are usually observed among younger women (<25 years) because of biological vulnerability (such as immature ectopic tissue on the cervix) and sexual behaviour which makes them prone to the growth of these pathogens (Lee, Tobin & Foley, 2006; Menezes et al., 2018). For example, amongst asymptomatic young HIV-negative South African women (<25 years), high rates of 33.5% and 11.1%, have been recorded for *CT* and *NG*, respectively (Menezes et al., 2018). Sexual behaviour was a significant risk factor, suggesting that more campaigns are needed to educate younger women and men about sexual and reproductive health (Menezes et al., 2018).

Notably, a high prevalence of *UP* (70.2%) and *MH* (36.6%) was observed, occurring in multiple infections. *UP* and *MH* are emerging pathobionts found in women both with healthy and unhealthy vaginal microbiota (Cox et al., 2016; Rumyantseva et al., 2019; Waites, Katz & Schelonka, 2005). The prevalence of *UP* is between 40–80%, while *MH* ranges between 21–53% in cervical/vaginal specimens of sexually active women (Cox et al., 2016; Waites, Katz & Schelonka, 2005). Previous studies have reported an association of these pathogens with bacterial vaginosis (BV) and STI, such as *MG*, and *CT* (Marovt et al., 2015). Rumyantseva and colleagues (2019) reported a significantly higher prevalence of *UP* (73.4%) among women with BV compared to women with normal bacterial flora (49.4%) or aerobic vaginitis (28.4%) (Rumyantseva et al., 2019). *MH* is considered BV-associated bacteria and has been reported to have a significantly higher bacterial load in women with BV compared to women without BV (Rumyantseva et al., 2019; Sha et al., 2005). Women with BV are reported to have high vaginal pH (>4.5) which is a favourable vaginal environment for pathogenic organisms (Kaambo et al., 2018). Additionally, women with detectable *MH* were often found to be co-infected with *Gardnerella vaginalis*, and such co-infection has been demonstrated in 60.7% of BV-positive women compared to BV-negative women (8.8%), which demonstrate a possible interaction between these pathogens (Cox et al., 2016). The transmission of either *MH* or *Gardnerella vaginalis* could activate the growth of the other, which may promote or contribute to the progression of BV (Cox et al., 2016). Verteramo and colleagues reported *UP* and *MH* as opportunistic pathogens of the lower female genital tract (Verteramo et al., 2009). However, The European STI guidelines do not recommend routine screening and treatment for these pathogens (Horner et al., 2018).

In this study, more than half of the study participants harboured multiple infections with sexually transmitted pathogens (52.7%). Multiple infections are reported to have a negative impact on the treatment of STIs and regarded as a risk factor for cervical cancer (Magaña Contreras et al., 2015)**.** Of the multiple infections, the co-infection of *UP*/HR-HPV occurred at a rate of 21.3% women, similar to that reported among sexually active women attending the outpatient clinic for routine cervical cancer screening (Parthenis et al., 2018). Moreover, a study among reproductive-age women from Gambia reported that 50% of women with HPV infection were co-infected with *UP* (Bah Camara et al., 2018). Women with detectable *UP* are found to have high levels of inflammatory cytokines, a biological co-factor that may increase the probability of persistent HPV infection and development of precancerous lesions (Biernat-Sudolska et al., 2011; Lobao et al., 2017; Roeters et al., 2010)**.** Similarly, the prevalence of *UP* was significantly 2-fold higher in women with high-grade squamous intraepithelial lesions (57.5%) compared to women with normal cytology (21.3%) (Farag et al., 2013)**.** The interaction of HR-HPV with *UP* demonstrates the need to screen for these pathogens as they may play a significant role in initiating the development of cervical cancer lesions.

The significant association of viral STIs (HSV-2 & HIV infection) with HR-HPV infection has been previously observed in other studies (Li & Wen, 2017; Taku et al., 2020). HSV-2 increases the odds of acquiring other STIs (such as *NG*) and is considered as the significant co-factor for HPV in the development of cervical cancer (Li & Wen, 2017; Smith et al., 2002a; Venkatesh et al., 2011). Women positive for HSV-2 and HIV infection were reported to have cervicovaginal inflammation and harbour a high diversity of microbes (Keller et al., 2019). These viral STIs have been found as independent risk factors of cervical cancer diseases. For example, a case-control study showed that HIV-positive women had a significantly increased prevalence of abnormal cytology (13.0%) compared to HIV-negative women (5.0%) (Suehiro et al., 2020). Similarly, the presence of HSV-2 infection was 5-fold higher in women with cervical intraepithelial neoplasia and squamous cell carcinoma compared to those with normal cervical cytology (Zhao et al., 2012)**.** Moreover, the co-infection of HPV/HSV-2 was significantly associated with cervical cancer lesions and cervical cancer than healthy women suggesting that this co-infection could be involved in the progression of cervical cancer (Zhao et al., 2012)**.** The findings highlight the need to consider awareness and educational programmes about the risk of these viruses in order to help reduce their outcome.

With South Africa having a high burden of STIs, particularly among asymptomatic women, an effective strategy to diagnose and treat STIs is needed. The high prevalence of HPV observed in this population confirms the need for HPV vaccination. Syndromic management policy has been reported to have low specificity and sensitivity in identifying the most common STIs, such as *NG* and *CT* (Maina, Kimani & Anzala, 2016; Marx et al., 2010). The syndromic management approach may not be good enough to control STIs when utilized alone as it results in a high STI prevalence of undiagnosed infections that may facilitate the transmission of HIV infection (Ward & Rönn, 2010). The high prevalence of STIs in this region encourages the need to implement diagnostic STI screening tests as the potential strategy to effectively decrease the burden of STI since the majority of STI-positive women are asymptomatic and remain untreated (Barnabas et al., 2018)**.** The screening will be beneficial not only for asymptomatic women but for those with symptoms in the general population or high-risk populations and facilitate receipt of appropriate treatment. Furthermore, considering the high prevalence of HR-HPV observed in this population, STI screening would be of assistance as it will help to reduce the burden of sexually transmitted pathogens that could potentially promote the development of persistence and cervical cancer lesions (Yong, 2017).

We acknowledge that the study had some potential limitations including small sample size, thus the results of this study cannot be regarded as the representative sample for the whole population of rural Eastern Cape Province. Also, the study was designed for HPV screening and this may result to a potential sampling bias in the context of sexually transmitted infections. Furthermore, in this study we depended on self-reported questionnaire for some data such as vaginal discharge, frequency of vaginal discharge and sexual behaviour. Therefore, this information may also introduce bias during the collection of participant information and analysis.

## Conclusion

A high prevalence of sexually transmitted pathogens, particularly *TV*, *UP*, and *MH* was documented in this rural community. HSV-2 and HIV were co-factors strongly associated with HR-HPV infection. The high prevalence of these pathogens underscores the need to revise the syndromic management policy by implementing effective strategies of prevention, screening tests, and management for sexually transmitted pathogens. The study also highlights the need to encourage routine screening of STIs for all women screened for cervical cancer. The high prevalence of HPV emphasizes the ongoing need for HPV vaccination.

##  Supplemental Information

10.7717/peerj.10793/supp-1 Supplemental Information 1 STI Raw Data

10.7717/peerj.10793/supp-2 Supplemental Information 2 Data on Symptoms

10.7717/peerj.10793/supp-3 Supplemental Information 3 The association of human papillomavirus infection and behavioural factors

10.7717/peerj.10793/supp-4 Supplemental Information 4 The association of sexually tranmitted infections and behavioural factors

## Abbreviations

STIs: sexually transmitted infections
BV: bacterial vaginosis
TP: *Treponema pallidum*
CT: *Chlamydia trachomatis*
HSV-2: Herpes simplex virus-2
MG: *Mycoplasma genitalium*
MH: *Mycoplasma hominis*
NG: *Neisseria gonorrhoeae*
TV: *Trichomonas vaginalis*
UP: Ureaplasma spp
HR-HPV: high-risk human papillomavirus

## Additional Information and Declarations

### Data Availability

The following information was supplied regarding data availability: The raw data are available in the Supplemental Files.
