# Supplementary material for: Detection of sexually transmitted pathogens and co-infection with human papillomavirus in women residing in rural Eastern Cape, South Africa
Source: PeerJ. 2021 Mar 3;9:e10793. doi: 10.7717/peerj.10793 (PMC7936566; doi:10.7717/peerj.10793)
Supplement: Supplemental Information 3 [file peerj-09-10793-s003.docx]

**Supplemental Table** 2: The association of human papillomavirus infection and behavioural factors

|  | **HR-HPV prevalence** | **Univariate analysis** |  | **Multivariate analysis** | |
| --- | --- | --- | --- | --- | --- |
| **Variables** | % (n/N) | **OR (95%CI)** | **p-value** | **OR (95%CI)** | **p-value** |
| Age in years: median-45 years (IQR:38-53) | | |  |  |  |
| **HIV infection** | |  |  |  |  |
| Negative | 25.4% (32/126) | Ref | Ref | Ref | Ref |
| Positive | 43.0% (34/79) | 2.22 (1.219-4.042) | **0.009** | 1.95 (0.790-4.827) | 0.147 |
| **Age category** | |  |  |  |  |
| 30-39 years | 40.6% (28/69) |  |  | Ref | Ref |
| 40-49 years | 37.3% (22/59) | 0.87 (0.426-1.777) | 0.704 | 1.28 (0.469-3.522) | 0.627 |
| ≥50 years | 20.8% (16/77) | 0.38 (0.185-0.797) | **0.010** | 0.81 (.266-2.482) | 0.717 |
| **Lifetime sexual partners** | |  |  |  |  |
| 1 | 16.1% (5/31) |  |  | Ref | Ref |
| 2 | 31.2% (19/61) | 2.35 (0.783-7.065) | 0.127 | 0.94 (0.150-5.932) | 0.951 |
| ≥3 | 37.2% (42/113) | 3.08 (1.098-8.619) | **0.033** | 1.42 (.235- 8.583) | 0.702 |
| **Sexual partners in 12 months** | |  |  |  |  |
| 0 | 24.1% (13/54) | Ref | Ref |  |  |
| ≥1 | 35.1% (53/151) | 1.71 (0.841-3.461) | 0.139 |  |  |
| **Sexual partners in the last month** | | |  |  |  |
| 0 | 28.2% (24/85) | Ref | Ref |  |  |
| ≥1 | 35.0% (42/120) | 1.37 (0.749-2.502) | 0.308 |  |  |
| **Used condoms during last sexual intercourse** | | |  |  |  |
| No | 31.0% (39/126) | Ref | Ref |  |  |
| Yes | 34.2% (26/76) | 1.16 (0.633-2.126) | 0.631 |  |  |
| **Frequency of vaginal sex past 1 month** | | |  |  |  |
| 0 | 30.7% (31/101) | Ref | Ref |  |  |
| 1-3 | 29.4% (20/68) | 0.94 (0.481-1.841) | 0.859 |  |  |
| ≥4 | 40.0% (14/35) | 1.50 (0.678-3.342) | 0.315 |  |  |
| **Vaginal discharge (self-reported)** | | |  |  |  |
| No | 31.6% (30/95) | Ref | Ref |  |  |
| Yes | 32.7% (36/110) | 1.05 (0.586-1.898) | 0.861 |  |  |
| **Frequency of vaginal discharge** | | |  |  |  |
| Current/last week | 43.2% (16/37) |  | Ref |  | Ref |
| More than a week and less than 6 months | 34.8% (8/23) | 0.70 (0.239-2.055) | 0.516 | 0.76 (0.246-2.316) | 0.624 |
| More than or equal to 6 months | 22.5% (11/49) | 0.38 (0.149-0.967) | **0.042** | 0.38 (0.141-1.029) | 0.057 |
| **Using any contraception with current partner** | | |  |  |  |
| No | 30.8% (37/120) | ref | ref |  |  |
| Yes | 35.4% (29/82) | 1.23 (0.676-2.227) | 0.500 |  |  |

**HR-HPV**: high-risk human papillomavirus, **OR**: odds ratio, **CI**: confidence intervals, **ref**: reference**, Highlighted values**; significant p-value
