# Supplementary material for: Detection of sexually transmitted pathogens and co-infection with human papillomavirus in women residing in rural Eastern Cape, South Africa
Source: PeerJ. 2021 Mar 3;9:e10793. doi: 10.7717/peerj.10793 (PMC7936566; doi:10.7717/peerj.10793)
Supplement: Supplemental Information 4 [file peerj-09-10793-s004.docx]

**Supplemental Table** 1: The association of sexually transmitted infections and behavioural factors

|  | **STD prevalence** |  | **Univariate analysis** |  |
| --- | --- | --- | --- | --- |
| **Variables** | % (n/N) |  | OR (95%CI) | p-value |
| Age in years: median-45 years (IQR:38-53) |  |  |  |  |
| **HIV infection** |  |  |  |  |
| Negative | 21.4% (27/126) |  | Ref | Ref |
| Positive | 25.3% (20/79) |  | 1.24 (0. 641-2.410) | 0.520 |
| **Age category** |  |  |  |  |
| 30-39 years | 31.9% (22/69) |  | Ref | Ref |
| 40-49 years | 17.0% (10/59) |  | 0.44 (0.187-1.018) | 0.055 |
| ≥50 years | 19.5% (15/77) |  | 0.52 (0.242 -1.103) | 0.088 |
| **Lifetime sexual partners** |  |  |  |  |
| 1 | 9.7% (3/31) |  | Ref | Ref |
| 2 | 19.7% (12/61) |  | 2.29 (0.594-8.796) | 0.229 |
| ≥3 | 28.3% (32/113) |  | 3.69 (1.047-12.986) | **0.042** |
| **Sexual partners in 12 months** |  |  |  |  |
| 0 | 22.2% (12/54) |  | Ref | Ref |
| ≥1 | 23.2% (35/151) |  | 1.06 (0.501-2.224) | 0.886 |
| **Sexual partners in the last month** |  |  |  |  |
| 0 | 24.7% (21/85) |  | Ref | Ref |
| ≥1 | 21.7% (26/120) |  | 0.84 (0.437-1.626) | 0.61 |
| **Used condoms during last sexual intercourse** |  |  |  |  |
| No | 23.8% (30/126) |  | Ref | Ref |
| Yes | 22.4% (17/76) |  | 0. 92 (0.468-1.816) | 0.814 |
| **Frequency of vaginal sex past 1 month** |  |  |  |  |
| 0 | 23.8% (24/101) |  | Ref | Ref |
| 1-3 | 26.5% (18/68) |  | 1.16 (0.569-2.343) | 0.690 |
| ≥4 | 14.3% (5/35) |  | 0. 54 (0.187-1.531) | 0.243 |
| **Vaginal discharge (self-reported)** |  |  |  |  |
| No | 20.0% (19/95) |  | Ref | ref |
| Yes | 25.5% (28/110) |  | 1.37 (0. 705-2.645) | 0.355 |
| **Frequency of vaginal discharge** |  |  |  |  |
| Current/last week | 18.9% (7/37) |  | Ref | ref |
| More than a week and less than 6 months | 26.1% (6/23) |  | 1.52 (0. 437-5.238) | 0.514 |
| More than or equal to 6 months | 28.6% (14/49 |  | 1.71 (0. 612-4.802) | 0.305 |
| **Using any contraception with current partner** |  |  |  |  |
| No | 19.2% (23/120) |  | Ref | ref |
| Yes | 29.3% (24/82) |  | 1.75 (0. 904-3.370) | 0.097 |
| **HR-HPV infection** |  |  |  |  |
| Negative | 20.9% (29/139) |  | Ref | ref |
| Positive | 27.3% (18/66) |  | 1.42 (0. 721-2.804) | 0.309 |

**Notes.**

**HR-HPV**: high-risk human papillomavirus, **OR**: odds ratio, **CI**: confidence intervals, **ref**: reference**, Highlighted values**; significant p-value

**STIs being analysed for this table**: *Chlamydia trachomatis*, *Herpes simplex virus-2*, *Neisseria gonorrhoeae* and *Trichomonas vaginalis*.
